# Sleep-disordered breathing and psychiatric comorbidities in narcolepsy: A 10-year retrospective study from Saudi Arabia

**DOI:** 10.1016/j.sleepx.2026.100199

**Published:** 2026-09-06

**Authors:** Mana Alshahrani, Nora Bedaiwi, Suhaila Aljawder, Riyad Allehebi, Ahmed Abdelbasit, Alyah A. Alshehri, Sumayyah I. Fallatah, Omaima T. Alomani, Lama A. Alhomayin, Talal N. AlHoshan

**Affiliations:** a Sleep Disorders Center, King Fahad Medical City, Riyadh Second Health Cluster, Riyadh, Saudi Arabia; b National Sleep Medicine Program, Ministry of Health, Riyadh, Saudi Arabia; c College of Medicine, Alfaisal University, Riyadh, Saudi Arabia; d Pediatric Neurology, King Fahad Medical City, Riyadh, Saudi Arabia; e Medical Cities, Ministry of Interior Medical Services, Riyadh, Saudi Arabia; f Family Medicine, Riyadh Second Health Cluster, Riyadh, Saudi Arabia

**Keywords:** Narcolepsy, Cataplexy, Obstructive sleep apnea, REM-OSA, Diagnostic delay, Saudi Arabia, Sleep medicine

## Abstract

**Background:**

Narcolepsy is a chronic disorder of central hypersomnia that is frequently under-recognized. Diagnostic delay, comorbid sleep-disordered breathing (SDB), and psychiatric comorbidities are well described internationally but remain poorly characterized in Saudi Arabia.

**Methods:**

We retrospectively reviewed all consecutive adult patients diagnosed with narcolepsy at the sleep disorders center of King Fahad Medical City (KFMC), a tertiary referral hospital in Riyadh, between January 2015 and April 2026. Diagnosis followed the third edition of the International Classification of Sleep Disorders (ICSD-3). Patients were classified as narcolepsy type 1 (NT1) when cataplexy was present and as narcolepsy type 2 (NT2) when MSLT criteria were met without cataplexy. Sleep breathing disorders and psychiatric conditions were assessed in all patients. Diagnostic delay was defined as the interval between patient-reported symptom onset and the year of formal diagnosis. Predictors of any sleep-disordered breathing (any-SDB: apnea–hypopnea index ≥ 5/h or REM-related OSA) and of diagnostic delay were examined with multivariable logistic and linear regression; the linear regression for diagnostic delay was performed on the natural-logarithm-transformed delay (log[delay + 1]) to satisfy the normality assumption. Cerebrospinal fluid hypocretin testing was not performed.

**Results:**

A total of 113 patients were analyzed (65 NT1 [57.5 %] and 48 NT2 [42.5 %]; 39 women [34.5 %]; median age 27 years [IQR 20–34]). Median body mass index (BMI) was 29.0 kg/m^2^ (IQR 24.9–34.0). Median diagnostic delay was 5 years (IQR 2–10; range 0–28) and increased with age (Kruskal–Wallis H = 10.7, p = 0.030; Pearson r with age = 0.43, p < 0.001). Any-SDB was present in 52/113 (46.0 %); REM-related OSA in 46/110 (40.7 %). After adjustment for narcolepsy type, BMI, and Epworth Sleepiness Scale (ESS), female sex was independently less at risk for any-SDB (adjusted odds ratio [aOR] 0.34, 95 % CI 0.13–0.88, p = 0.026) and older age conferred increased risk (aOR 1.06 per year, 95 % CI 1.01–1.11, p = 0.023). In the multivariable linear model for log-transformed diagnostic delay (adjusted R^2^ = 0.119), age was the only independent predictor (β = +0.020 per year on the log scale, equivalent to a 2.0 % increase in delay per year of age; 95 % CI + 0.002 to +0.038, p = 0.032). ESS independently predicted dichotomized delayed diagnosis (≥5 years) (aOR 1.15 per ESS point, 95 % CI 1.03–1.29, p = 0.013). Cataplexy was, by definition, present in all NT1 patients (100 %) and inabsent of all NT2 patients. Sleep paralysis (61.5 % vs 33.3 %, p = 0.003) and hypnagogic/hypnopompic hallucinations (50.8 % vs 16.7 %, p < 0.001) were significantly more common in NT1. Modafinil was the dominant wake-promoting agent (88.5 %); antidepressant prescribing was concentrated in NT1 (any-antidepressant 73.8 % vs 22.9 %, p < 0.001). Major depressive disorder (16.8 %) and generalized anxiety disorder (9.7 %) showed no subtype difference.

**Conclusions:**

In this Saudi tertiary-center cohort, narcolepsy was associated with a substantial diagnostic delay that increased with age at presentation, and with a high prevalence of comorbid sleep-disordered breathing, in which female sex and younger age were independent predictors of lower risk for SDB. The dataset supports stronger advocacy for early MSLT testing in adult patients with chronic excessive daytime sleepiness, and for systematic screening of psychiatric comorbidity at narcolepsy diagnosis.

## 1 Introduction

Narcolepsy is a chronic, neurologic disorder of REM-sleep regulation characterized by excessive daytime sleepiness (EDS), cataplexy, hypnagogic or hypnopompic hallucinations, sleep paralysis, and disrupted nocturnal sleep [1,2]. Global prevalence is estimated at 25–50 per 100 000 individuals with substantial geographic variability [3]. Despite these distinctive features, narcolepsy is consistently under-recognized, particularly outside high-resource sleep-medicine centers.

In Saudi Arabia, BaHammam and Alenezi previously reported a mean diagnostic delay of 8.4 years between symptom onset and formal diagnosis and observed that only 6.4 % of patients referred to a tertiary sleep center presented with the correct diagnosis [4]. The factors contributing to this delay include limited primary-care familiarity with narcolepsy, overlap with other causes of EDS, most prominently obstructive sleep apnea (OSA), and the requirement for specialized diagnostic testing (polysomnography [PSG] with multiple sleep latency testing [MSLT]) [5].

Obstructive sleep apnea is the most common sleep disorder worldwide, affecting an estimated 9–38 % of adults and exhibiting a male predominance [6]. In a large Saudi population-based study, Wali and colleagues estimated overall OSA prevalence at 8.8 % (12.8 % in men, 5.1 % in women), with obesity, hypertension, and male sex as the dominant risk factors [7]. Because EDS is a cardinal symptom of both OSA and narcolepsy, the two conditions are frequently confounded clinically, and patients with narcolepsy may be initially managed for presumptive OSA before the correct diagnosis is established [5,6].

Recent data suggest that OSA, particularly REM-related OSA (REM-OSA), is over-represented among adults with narcolepsy [5,8]. In a recent Saudi study, Dhafer and colleagues reported OSA in 55.8 % of narcoleptic patients and REM-OSA in 26.4 % [9]. Despite this growing evidence base, no comprehensive Saudi study has yet integrated demographics, symptom profile, sleep architecture, sleep-disordered breathing, psychiatric comorbidity, and pharmacological management in a single narcolepsy cohort. Such integrated data are essential for informing local clinical guidelines, accelerating accurate diagnosis, and rationalizing treatment.

We therefore conducted a retrospective cohort study at King Fahad Medical City, a tertiary referral sleep-medicine centre, to (i) characterise the demographic and clinical profile of patients with narcolepsy, (ii) quantify diagnostic delay and its predictors, (iii) describe sleep-disordered breathing and its independent predictors after adjustment for confounders, and (iv) describe psychiatric comorbidities and the current pharmacological treatement landscape in this population.

## 2 Materials and methods

### 2.1 Study design and setting

This was a retrospective, single-center observational cohort study conducted at the Sleep Disorders Center of King Fahad Medical City (KFMC), a tertiary referral medical city in Riyadh, Saudi Arabia. The study period extended from January 2015 to April 2026. The protocol was approved by the KFMC Institutional Review Board (IRB approval no. 26-131); the requirement for informed consent was waived in view of the retrospective design and the use of fully de-identified data.

### 2.2 Eligibility criteria

Inclusion criteria were (i) consecutive adult with a clinical diagnosis of narcolepsy meeting the third International Classification of Sleep Disorders (ICSD-3) criteria; (ii) availability of an overnight diagnostic in-laboratory polysomnogram (PSG) without continuous positive airway pressure (CPAP) titration, with sufficient data for sleep-staging and respiratory analysis; and (iii) availability of a same-admission multiple sleep latency test (MSLT) interpreted by a sleep-medicine consultant.

Exclusion criteria were (i) incomplete clinical or PSG records, (ii) idiopathic hypersomnia, Kleine–Levin syndrome, or secondary hypersomnia (e.g., shift-work, sleep-deprivation), (iii) other central disorders of hypersomnolence inconsistent with narcolepsy on MSLT, (iv) major neurologic or cardiopulmonary disease likely to alter sleep architecture or breathing, and (v) use of REM-suppressing or respiratory-depressant medications that were not appropriately withheld prior to PSG/MSLT.

All REM-suppressing or respiratory-depressant medications (including selective serotonin reuptake inhibitors, serotonin–norepinephrine reuptake inhibitors, tricyclic antidepressants, and centrally acting sedatives) were withheld for at least 14 days (two weeks) prior to PSG and MSLT in accordance with standard sleep-laboratory protocol; patients in whom the washout could not be safely completed were excluded from the analysis.

### 2.3 Case ascertainment and classification

Patients were identified by querying KFMC's electronic medical record (EMR) and PSG database using diagnostic codes and clinic letters. Narcolepsy was sub-classified per ICSD-3 as narcolepsy type 1 (NT1) when clinically observed or convincingly reported cataplexy was present, and as narcolepsy type 2 (NT2) when MSLT criteria for narcolepsy were met without cataplexy. Cerebrospinal fluid (CSF) hypocretin-1 measurement and HLA-DQB1*06:02 genotyping were not performed at our center; the absence of these biomarker confirmations is addressed in the study Limitations.

### 2.4 Variables and definitions

The following variables were abstracted: demographics (age, sex, body mass index [BMI], occupation); symptom domains (cataplexy, sleep paralysis, hypnagogic/hypnopompic hallucinations, disrupted nocturnal sleep) coded as present/absent; subjective sleepiness assessed by the Epworth Sleepiness Scale (ESS); PSG parameters (apnea–hypopnea index [AHI; events/hour], REM-AHI, sleep efficiency); diagnostic delay calculated as (year of formal diagnosis − patient-reported year of symptom onset); physician-diagnosed psychiatric comorbidities (major depressive disorder [MDD], generalized anxiety disorder [GAD], insomnia disorder, restless legs syndrome [RLS], parasomnia) and pharmacological prescriptions (modafinil, methylphenidate, venlafaxine, fluoxetine, escitalopram, imipramine, clomipramine).

Psychiatric diagnoses, specifically major depressive disorder and generalized anxiety disorder, were ascertained from formal psychiatric evaluation and physician-documented diagnoses recorded in the EMR, supported by the corresponding ICD-10 diagnostic codes. Patients with isolated psychiatric symptoms but without a formal physician-documented diagnosis or an ICD-10 code were not coded as having the corresponding psychiatric disorder.

OSA was defined as a baseline diagnostic AHI ≥ 5 events/hour on the in-laboratory PSG. REM-OSA was defined using the criteria of Mokhlesi and Punjabi (as adopted by Dhafer and colleagues): REM-AHI ≥ 5 events/hour, a REM-AHI/NREM-AHI ratio > 2, and ≥ 30 min of REM sleep [9,10]. Any sleep-disordered breathing (any-SDB) was defined as OSA or REM-OSA. PSG followed the American Academy of Sleep Medicine (AASM) scoring rules current at the time of testing. All PSGs included in this analysis were diagnostic studies; split-night and titration-only studies were excluded.

### 2.5 Statistical analysis

Continuous variables were screened for normality with the Shapiro–Wilk test and visual inspection. Variables not approximating a normal distribution were summarised as median (interquartile range [IQR]) and compared between groups with the Mann–Whitney U or Kruskal–Wallis tests; approximately normal variables were summarised as mean ± standard deviation (SD) and compared with the Welch *t*-test. Categorical variables were summarised as n (%) and compared with the Pearson chi-squared test; Fisher's exact test was substituted where any expected cell count was less than five.

Because diagnostic delay was strongly right-skewed (median 5 years, range 0–28 years), the multivariable linear regression for diagnostic delay was performed on the natural-logarithm-transformed delay (log[delay + 1]) to satisfy the assumption of approximately normally distributed residuals. The transformation improved residual normality (Shapiro–Wilk p increased from 0.033 on the raw scale to 0.090 on the log scale). Coefficients are reported on the log scale together with their multiplicative interpretation on the natural scale.

Predictors of any-SDB were examined with multivariable logistic regression, with a priori covariates of age, sex, BMI, narcolepsy type, and ESS. Predictors of (log-transformed) continuous diagnostic delay were examined with multivariable linear regression with the same a priori covariates; predictors of dichotomized delayed diagnosis (≥5 years) were examined with logistic regression. Variance-inflation factors were computed to screen for multicollinearity. A complete-case approach was used; the denominator for each test is reported. Sensitivity analyses were planned for (i) per-test denominators of categorical variables and (ii) exclusion of four patients with chart notes indicating diagnostic uncertainty (“? narcolepsy” or “MSLT suggestive of sleep deprivation”). Statistical significance was set a priori at two-sided α = 0.05. Analyses were conducted using Python 3 (pandas, scipy, statsmodels).

## 3 Results

### 3.1 Patient cohort

**Fig. 1:**
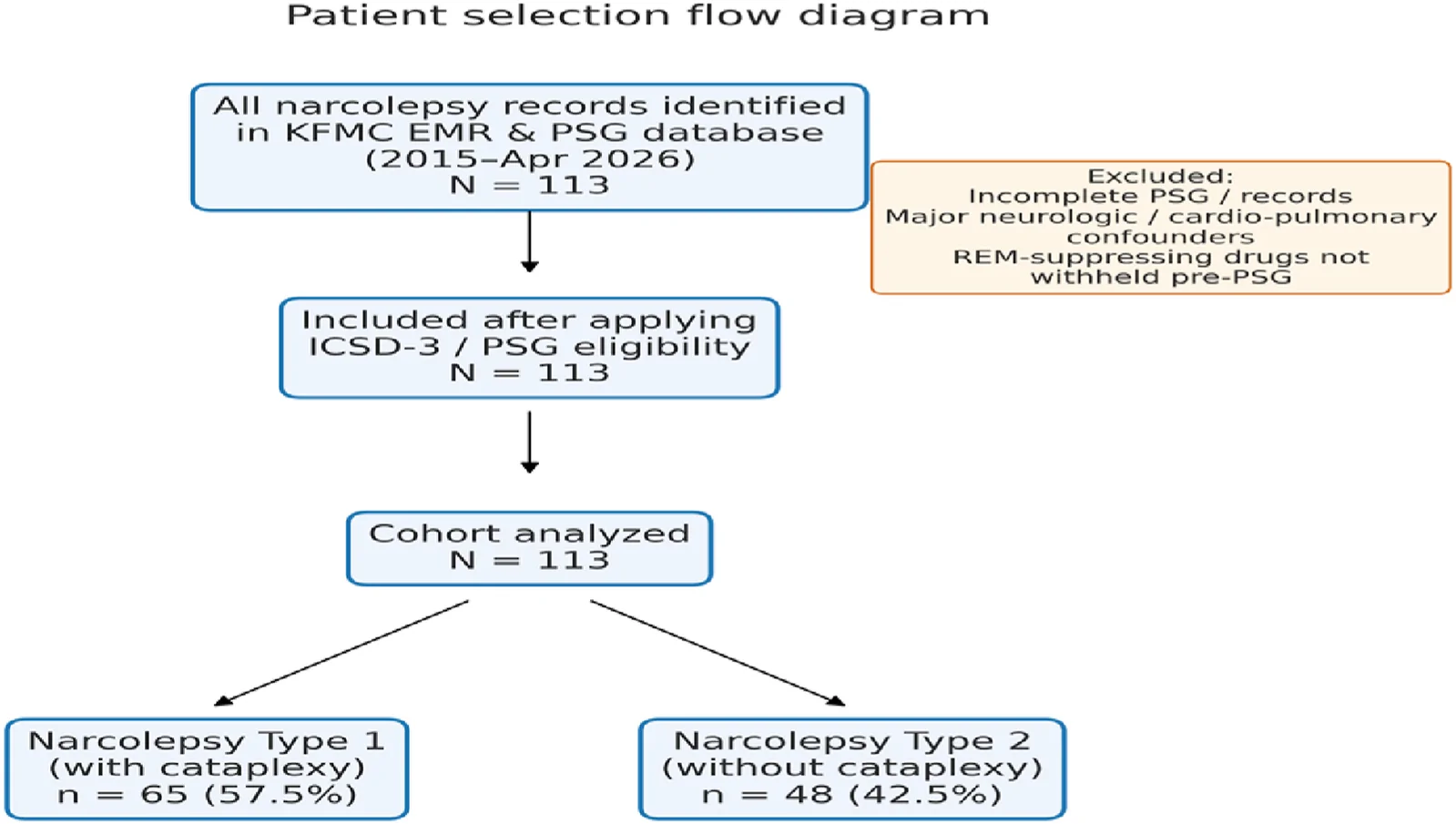
Patient selection flow diagram Identification of patients meeting eligibility criteria during the study window (January 2015 – April 2026).

From January 2015 to April 2026, 113 patients met the inclusion criteria (Fig. 1). NT1 was diagnosed in 65 (57.5 %) and NT2 in 48 (42.5 %). 39 patients (34.5 %) were women, and 15 (13.3 %) were pediatric (<18 years). Median age was 27 years (IQR 20–34, range 14–54); median BMI was 29.0 kg/m^2^ (IQR 24.9–34.0). Median ESS was 17 (IQR 14–20). Baseline characteristics for the whole cohort are presented in Table 1; differences by narcolepsy subtype are summarised in Table 2.

**Table 1:** Baseline characteristics of the whole cohort (N = 113).

Characteristic	Value n (%)
Age, years, median (IQR)	27 (20–34)
Female sex, n (%)	39 (34.5)
BMI, kg/m^2^, median (IQR)	29.0 (24.9–34.0)
ESS, median (IQR)	17 (14–20)
NT1 (with cataplexy), n (%)	65 (57.5)
NT2 (without cataplexy), n (%)	48 (42.5)
Sleep paralysis, n (%)	56/112 (50.0)
Hypnagogic/hypnopompic hallucinations, n (%)	41 (36.3)
Disrupted nocturnal sleep, n (%)	50 (44.2)
AHI, events/h, median (IQR)	4.4 (1.7–7.7)
REM-AHI, events/h, median (IQR)	10.7 (3.8–20.6)
Sleep efficiency, %, median (IQR)	85 (78–91)
Any sleep-disordered breathing, n (%)	52 (46.0)
Diagnostic delay, years, median (IQR)	5 (2–10)
Major depressive disorder, n (%)	19 (16.8)
Generalized anxiety disorder, n (%)	11 (9.7)
Insomnia disorder, n (%)	11 (9.7)
Restless legs syndrome, n (%)	1 (0.9)
Modafinil exposure, n (%)	100 (88.5)
Methylphenidate exposure, n (%)	18 (15.9)
Any antidepressant exposure, n (%)	59 (52.2)

AHI = apnea–hypopnea index; BMI = body mass index; ESS = Epworth Sleepiness Scale; IQR = interquartile range; NT1 = narcolepsy type 1; NT2 = narcolepsy type 2.

**Table 2:** Narcolepsy type 1 versus type 2 (per-test denominators).

Variable	NT1 (n = 65)	NT2 (n = 48)	p
Age (years)	26.0 (20.0–33.0)	27.0 (22.2–34.2)	0.708
BMI (kg/m^2^)	29.0 (25.0–34.8)	28.0 (24.0–31.1)	0.150
ESS	17.0 (14.0–21.0)	16.0 (12.0–19.0)	0.228
Female sex	24/65 (36.9 %)	15/48 (31.2 %)	0.531
Pediatric (<18 y)	10/65 (15.4 %)	5/48 (10.4 %)	0.442
Cataplexy	65/65 (100.0 %)	0/48 (0.0 %)	0.000
Sleep paralysis	40/64 (62.5 %)	16/48 (33.3 %)	0.002
Hallucinations	33/65 (50.8 %)	8/48 (16.7 %)	0.000
Interrupted sleep	33/65 (50.8 %)	17/48 (35.4 %)	0.104
OSA	26/63 (41.3 %)	18/47 (38.3 %)	0.753
REM-OSA	28/63 (44.4 %)	18/47 (38.3 %)	0.518
Any OSA	30/65 (46.2 %)	22/48 (45.8 %)	0.973
MDD	10/65 (15.4 %)	9/48 (18.8 %)	0.636
GAD	5/65 (7.7 %)	6/48 (12.5 %)	0.524
Insomnia	5/65 (7.7 %)	6/48 (12.5 %)	0.524
Modafinil	60/65 (92.3 %)	40/48 (83.3 %)	0.139
Methylphenidate	8/65 (12.3 %)	10/48 (20.8 %)	0.221
Venlafaxine	26/65 (40.0 %)	3/48 (6.2 %)	0.000
Fluoxetine	12/65 (18.5 %)	2/48 (4.2 %)	0.023
Escitalopram	7/65 (10.8 %)	3/48 (6.2 %)	0.513
Clomipramine	7/65 (10.8 %)	0/48 (0.0 %)	0.020
Any antidepressant	48/65 (73.8 %)	11/48 (22.9 %)	0.000
AHI	5.0 (1.6–7.3)	3.7 (2.0–7.9)	0.912
REM-AHI	11.0 (3.3–21.4)	10.2 (5.1–19.3)	0.982
Diagnostic delay (years)	4.0 (3.0–10.0)	6.0 (2.0–10.0)	0.865

Highlighted rows = p < 0.05. Per-test denominators: cells with missing values were excluded from each comparison. Cataplexy is, by definition under ICSD-3, present in all NT1 patients and absent in NT2 patients in this cohort. AHI and REM-AHI are reported as median (IQR) given non-normal distributions (Shapiro–Wilk p < 0.001).

### 3.2 Comparison of narcolepsy type 1 versus type 2

Significant differences between NT1 and NT2 were observed for the REM-intrusion symptoms (cataplexy, sleep paralysis, hypnagogic hallucinations) and for antidepressant prescribing (Table 2). Patient age, sex distribution, BMI, ESS, AHI, REM-AHI, sleep efficiency, diagnostic delay, sleep-disordered breathing, psychiatric comorbidities, and wake-promoting drug exposure did not differ significantly between subtypes.

### 3.3 Sleep architecture and sleep-disordered breathing

Sleep architecture data available for analysis were limited to sleep efficiency, AHI, and REM-AHI; stage percentages, total sleep time, and REM latency were not consistently abstracted in the EMR and therefore represent targets for prospective re-abstraction. Median sleep efficiency was preserved at 85 % overall (IQR 78–91 %) and did not differ between subtypes (Table 2).

Sleep-disordered breathing was present in 52/113 patients (46.0 %); 38.9 % of patients with PSG data met criteria for diagnostic OSA (44/113; missing in 3), and 40.7 % met criteria for REM-OSA (46/110). NT1 and NT2 had virtually identical rates of any-SDB (46.2 % vs 45.8 %, p = 0.973), OSA (40.0 % vs 37.5 %, p = 0.788), and REM-OSA (43.1 % vs 37.5 %, p = 0.551).

In multivariable logistic regression adjusting for narcolepsy type, BMI, age, and ESS (n = 99), female sex was independently less risk factor for any-SDB (adjusted odds ratio [aOR] 0.34, 95 % CI 0.13–0.86, p = 0.023), and older age independently increased the odds (aOR 1.05 per year, 95 % CI 1.00–1.11, p = 0.037). Narcolepsy type (aOR 1.22, p = 0.661), BMI (aOR 1.00, p = 0.949), and ESS (aOR 1.04, p = 0.346) showed no independent association (Table 3; Fig. 5). Consistent with the adjusted analysis, the unadjusted prevalence of OSA (50.0 % vs 17.9 %, p = 0.001), REM-OSA (50.0 % vs 23.1 %, p = 0.006), and any-SDB (55.4 % vs 28.2 %, p = 0.006) was markedly higher in men than in women (Fig. 6).

**Table 3:** Sleep-disordered breathing by narcolepsy subtype.

Variable	NT1 (n = 65)	NT2 (n = 48)	p
AHI, events/h, median (IQR)	5.0 (1.6–7.3)	3.7 (2.0–7.9)	0.912
REM-AHI, events/h, median (IQR)	11.0 (3.3–21.4)	10.2 (5.1–19.3)	0.982
Sleep efficiency, %, median (IQR)	85 (79–90)	85 (74–92)	0.639
OSA (AHI ≥ 5), n (%)	26/63 (41.3)	18/47 (38.3)	0.788
REM-OSA, n (%)	28/63 (44.4)	18/47 (38.3)	0.551
Any SDB, n (%)	30/65 (46.2)	22/48 (45.8)	0.973

### 3.4 Diagnostic delay

Diagnostic delay was calculable in 90 of 113 patients (79.6 %); 23 patients had missing data or had recall difficulties regarding the symptom-onset year. Median delay was 5 years (IQR 2–10, range 0–28); mean ± SD 6.6 ± 5.7 years. Delay increased monotonically with age stratum (Table 5a; Fig. 3): from 3.8 ± 2.4 years among patients ≤ 20 years to 12.2 ± 9.5 years among those 41–50 years. The one patient aged >50 years had a 10-year delay. Across strata, Kruskal–Wallis H = 10.74, p = 0.030 (one-way ANOVA F = 7.02, p < 0.001). The empirical cumulative distribution of diagnostic delay across the cohort is shown in Fig. 4. (see Table 4a, Table 4ba and b).

**Fig. 2:**
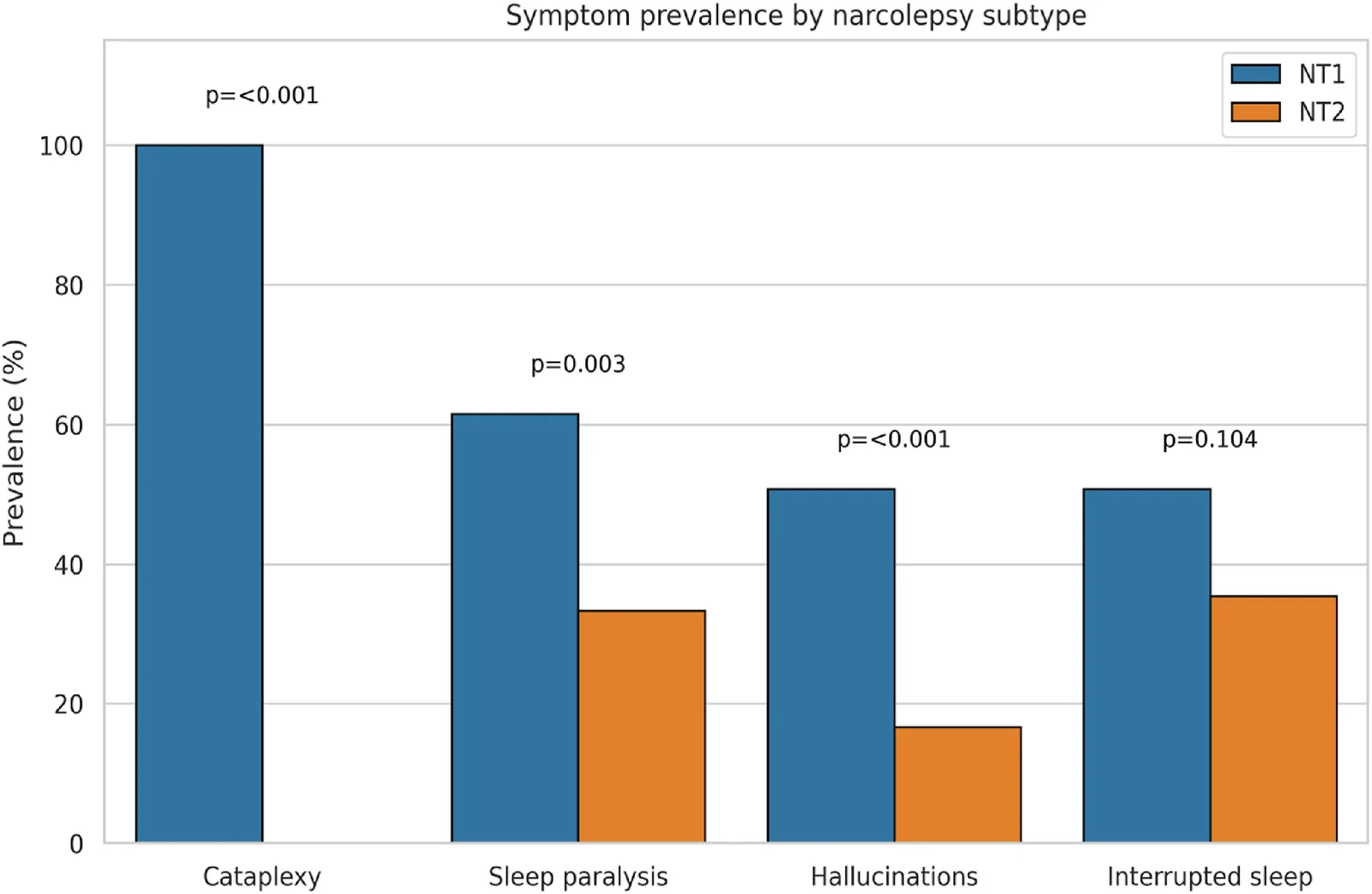
Symptom prevalence by narcolepsy subtype Cardinal narcoleptic symptoms by ICSD-3 subtype. p-values from Pearson chi-squared test.

**Fig. 3:**
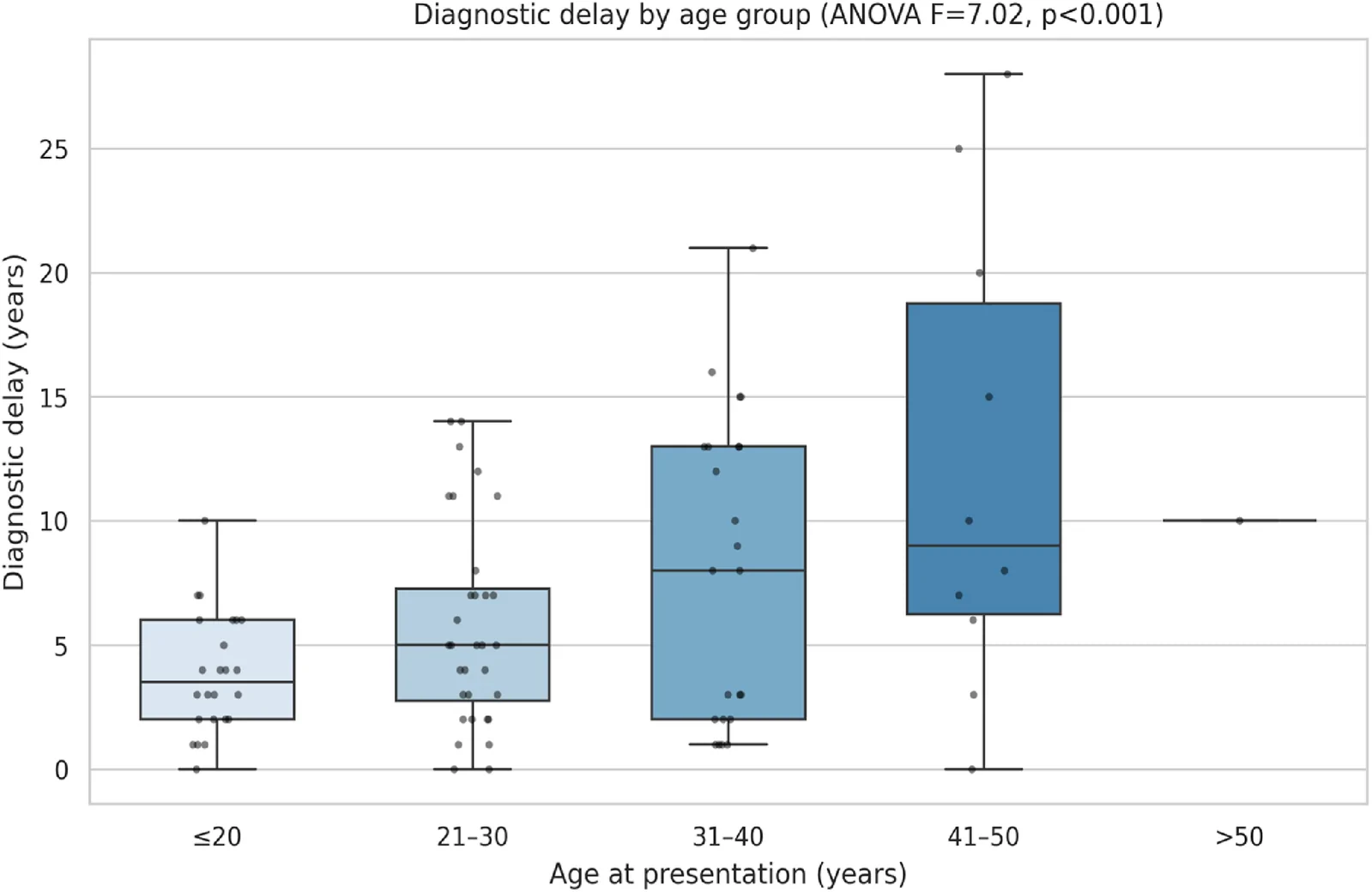
Diagnostic delay by age group Box-and-whisker plot of diagnostic delay across five age strata (boxes: median and IQR; whiskers: 1.5 × IQR; dots: individual patients).

**Fig. 4:**
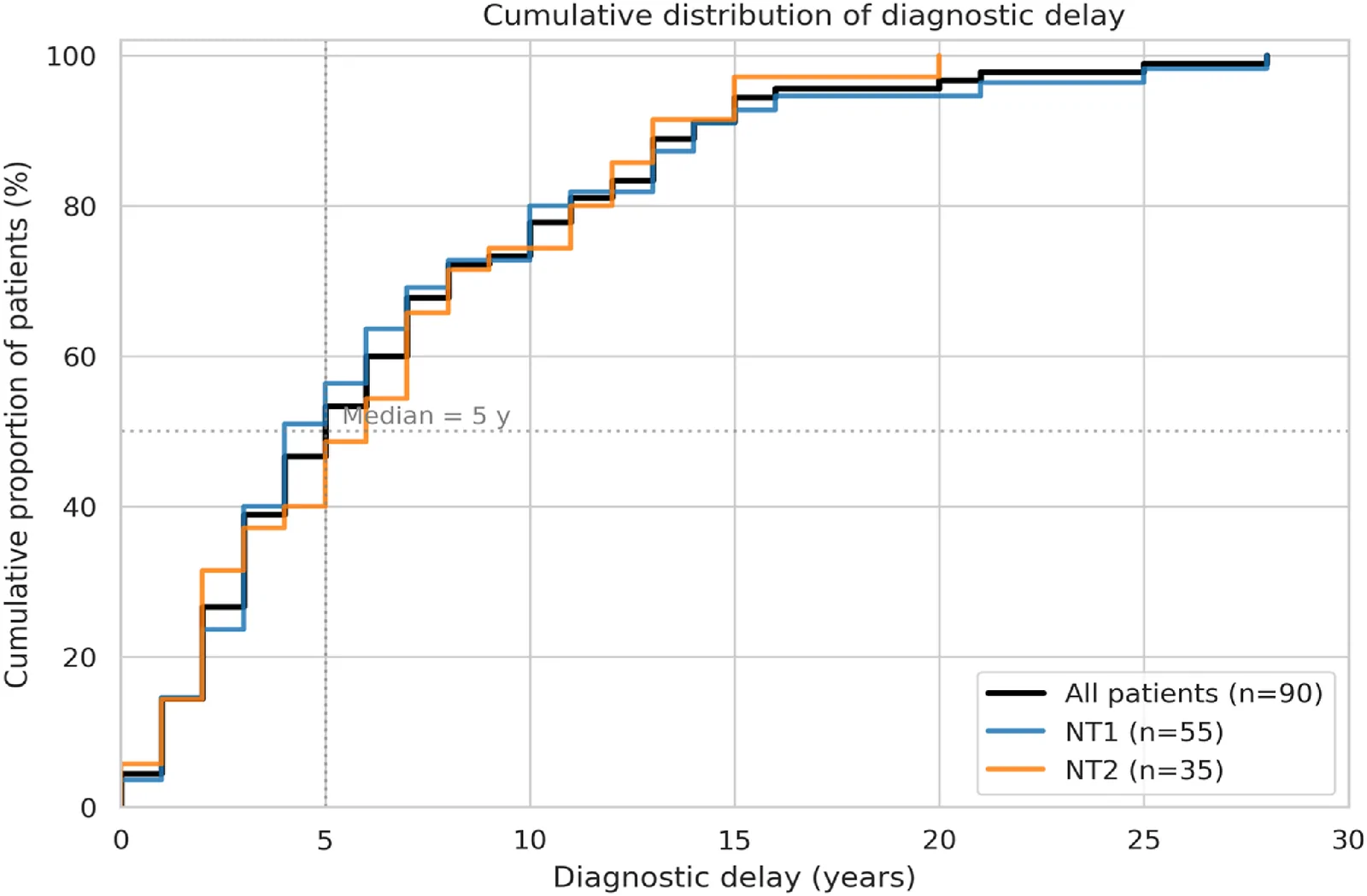
Cumulative distribution of diagnostic delay Empirical cumulative distribution function (cumulative frequency curve) of diagnostic delay across the cohort and by narcolepsy subtype (n = 90 with complete delay data). The y-axis shows the cumulative proportion of patients whose delay was at or below the value on the x-axis. The dashed reference line indicates the cohort median of 5 years.

**Fig. 5:**
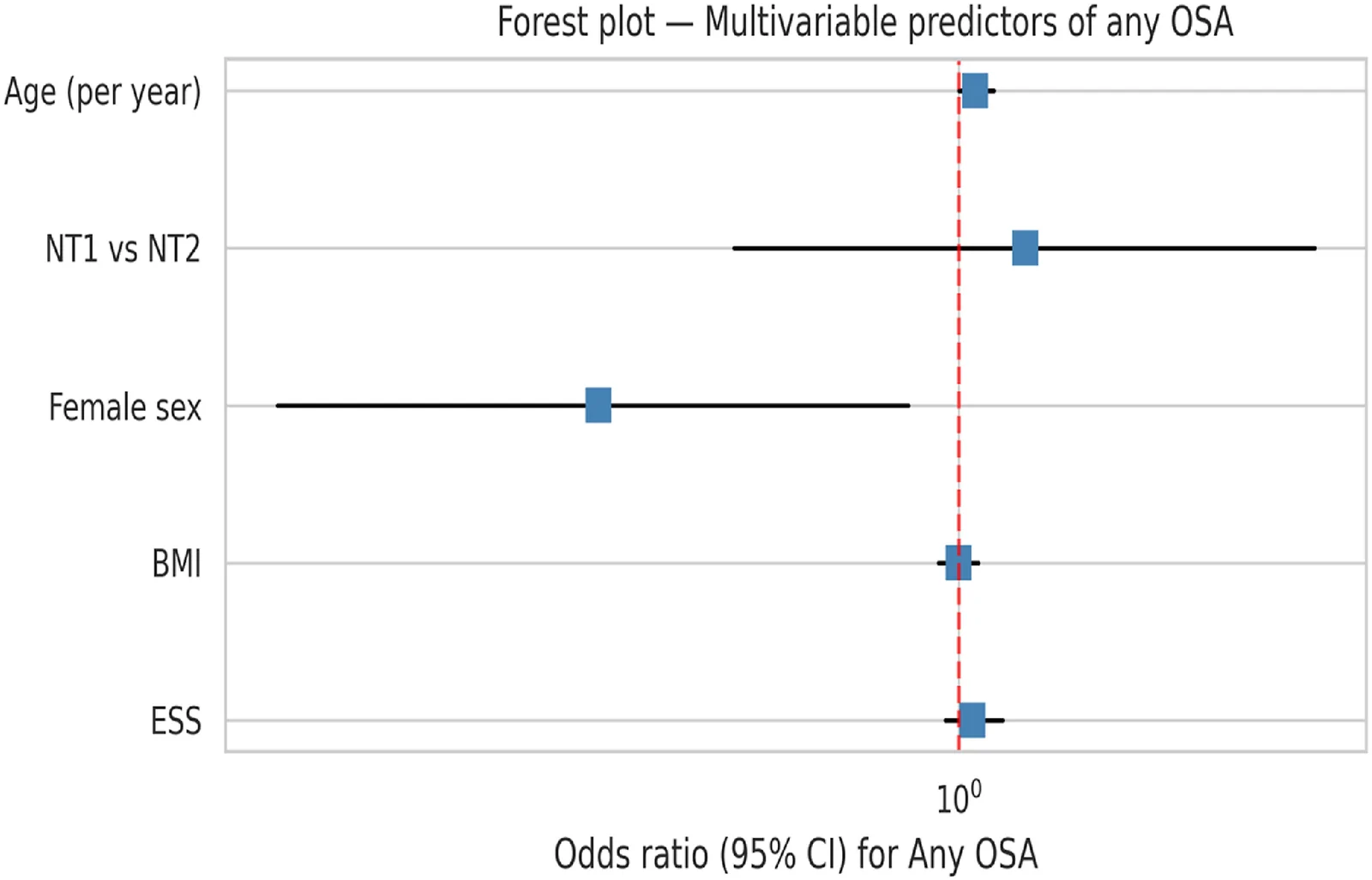
Forest plot: Multivariable predictors of any sleep-disordered breathing. Adjusted odds ratios with 95 % confidence intervals from multivariable logistic regression (n = 99; pseudo-R^2^ = 0.09; likelihood-ratio test p = 0.026). Reference categories: NT2 for narcolepsy subtype, male for sex. Female sex was independently protective (adjusted OR 0.34, 95 % CI 0.13–0.86, p = 0.023) and older age was an independent risk factor (adjusted OR 1.05 per year, 95 % CI 1.00–1.11, p = 0.037).

**Fig. 6:**
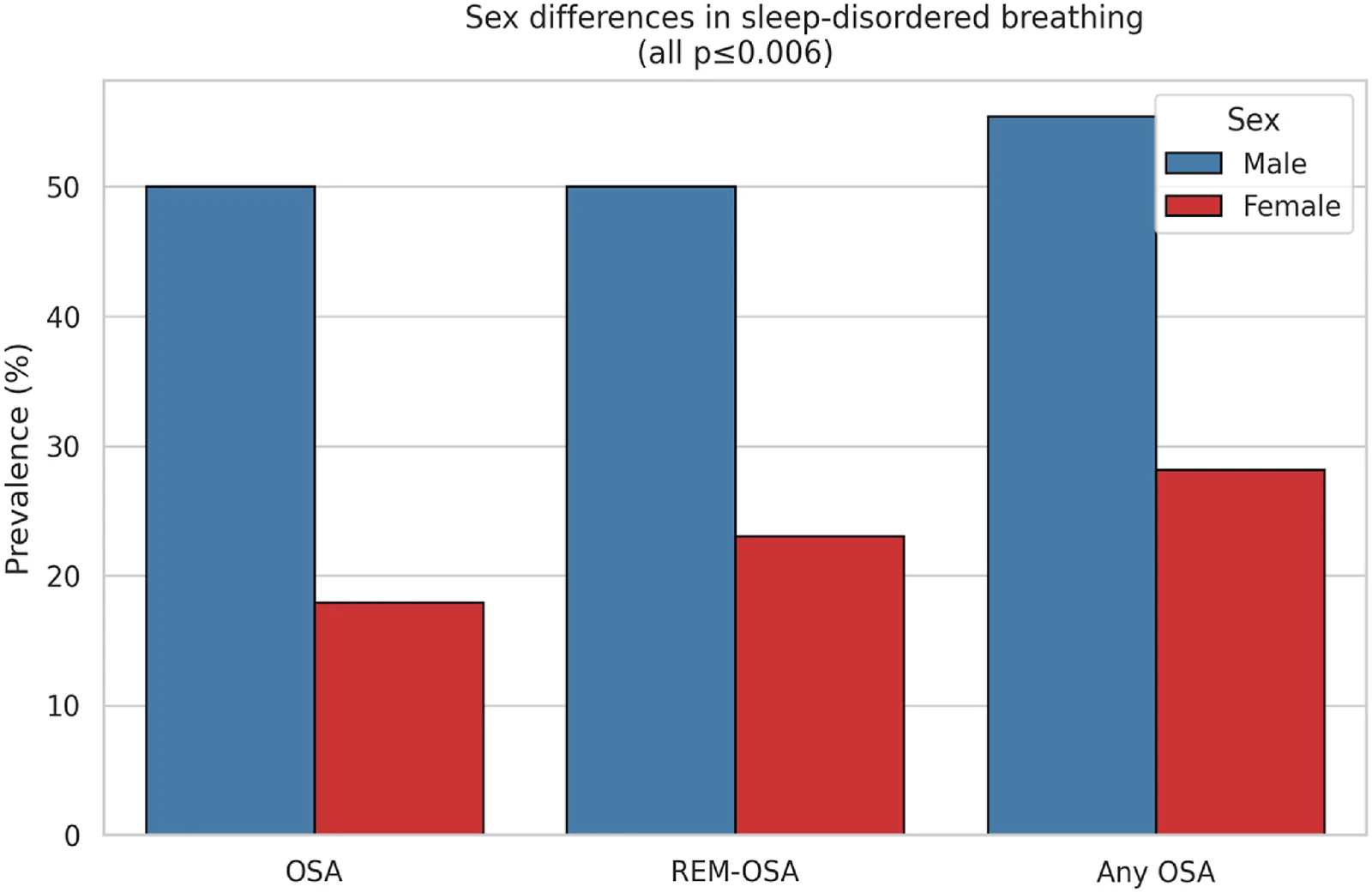
Sex differences in sleep-disordered breathing. Prevalence of obstructive sleep apnea (OSA, AHI ≥ 5/h), REM-related OSA, and any sleep-disordered breathing (any-SDB) among male and female patients. Men had a markedly higher prevalence across all three definitions (OSA 50.0 % vs 17.9 %, p = 0.001; REM-OSA 50.0 % vs 23.1 %, p = 0.006; any-SDB 55.4 % vs 28.2 %, p = 0.006).

**Table 4a:** Multivariable logistic regression: predictors of any sleep-disordered breathing.

Predictor	Adjusted odds ratio	95 % CI	p
Age (per year)	1.05	1.00–1.11	0.037
NT1 vs NT2	1.22	0.51–2.90	0.661
Female vs Male	0.34	0.13–0.86	0.023
BMI (per kg/m^2^)	1.00	0.94–1.06	0.949
ESS (per point)	1.04	0.96–1.14	0.346

Complete-case n = 99; pseudo-R^2^ = 0.09; Likelihood-ratio test p = 0.026.

**Table 4b:** Age-stratified sleep-disordered breathing and psychiatric comorbidities.

Age group (years)	n	Any SDB, n (%)	OSA (AHI ≥ 5/h), n (%)	REM-OSA, n (%)	AHI, median (IQR)	REM-AHI, median (IQR)	MDD, n (%)	GAD, n (%)
≤ 20	29	11 (37.9)	8/28 (28.6)	11/28 (39.3)	2.5 (1.0–5.5)	8.2 (2.0–18.7)	4 (13.8)	1 (3.4)
21–30	39	13 (33.3)	12/38 (31.6)	11/38 (28.9)	3.6 (1.7–6.6)	10.0 (4.3–16.2)	5 (12.8)	3 (7.7)
31–40	29	17 (58.6)	14/28 (50.0)	14/28 (50.0)	5.0 (2.1–13.0)	11.4 (3.9–24.9)	7 (24.1)	5 (17.2)
41–50	15	10 (66.7)	9 (60.0)	9 (60.0)	6.9 (2.4–8.9)	17.8 (7.1–26.9)	3 (20.0)	1 (6.7)
> 50	1	1 (100)	1 (100)	1 (100)	—	—	0 (0)	1 (100)
**Overall**	**113**	**52 (46.0)**	**44/110 (40.0)**	**46/110 (40.7)**	**4.4 (1.7–7.7)**	**10.7 (3.8–20.6)**	**19 (16.8)**	**11 (9.7)**

Table 4b. Age-stratified prevalence of sleep-disordered breathing (SDB), REM-related obstructive sleep apnea (REM-OSA), respiratory polysomnographic indices, and psychiatric comorbidities (n = 113). Denominators smaller than the age-stratum n reflect missing PSG data for a given variable. Trend across age strata for any-SDB: logistic-regression odds ratio 1.06 per year of age (95 % CI 1.02–1.11, p = 0.007). AHI = apnea–hypopnea index; GAD = generalized anxiety disorder; IQR = interquartile range; MDD = major depressive disorder; OSA = obstructive sleep apnea; SDB = sleep-disordered breathing.

Among univariate predictors, age was most strongly correlated with delay (Pearson r = 0.43, Spearman ρ = 0.35; both p < 0.001), and ESS was modestly correlated (ρ = 0.23, p = 0.036). REM-AHI showed a non-significant trend (ρ = 0.19, p = 0.089). In the multivariable linear model for log-transformed diagnostic delay (n = 85; adjusted R^2^ = 0.119, F p = 0.022) adjusting for age, sex, BMI, ESS, narcolepsy type, MDD, insomnia and any-SDB, age was the only independent predictor (β = +0.020 per year on the log scale, equivalent to a 2.0 % increase in delay per year of age; 95 % CI + 0.002 to +0.038, p = 0.032), with ESS showing a borderline effect (β = +0.031 per point on the log scale, equivalent to a 3.1 % increase per ESS point; p = 0.080). Regression on the raw (untransformed) delay yielded the same direction of effects but produced right-skewed residuals (Shapiro–Wilk p = 0.033); log-transformation normalized the residuals (Shapiro–Wilk p = 0.090).

When delay was dichotomized at the median (≥5 vs < 5 years), 48 patients were classified as delayed and 42 as early-diagnosed. In adjusted logistic regression, ESS was an independent risk factor for delayed diagnosis (aOR 1.15 per ESS point, 95 % CI 1.03–1.29, p = 0.013); age (aOR 1.05, p = 0.099) and NT1 (aOR 0.40, p = 0.093) approached but did not reach statistical significance.

An exploratory analysis examined whether the presence of cardinal narcoleptic symptoms shortened diagnostic delay. Patients with cataplexy did not differ from those without (median 4.0 vs 6.0 years, p = 0.865); patients with sleep paralysis (5.0 vs 4.0, p = 0.129), hallucinations (6.0 vs 5.0, p = 0.517), and disrupted nocturnal sleep (6.0 vs 4.0, p = 0.092) showed no significant difference. The absence of a “cataplexy advantage” indicates that even classical NT1 presentations may go unrecognized for years (see Table 5b).

**Table 5a:** Diagnostic delay by age stratum and narcolepsy subtype.

Age group (years)	Overall, mean ± SD (n)	NT1	NT2
≤ 20	3.8 ± 2.4 (24)	3.9 ± 2.5 (15)	3.7 ± 2.3 (9)
21–30	5.8 ± 4.1 (32)	5.9 ± 4.3 (18)	5.5 ± 4.0 (14)
31–40	8.0 ± 6.1 (23)	7.9 ± 6.8 (14)	8.3 ± 5.4 (9)
41–50	12.2 ± 9.5 (10)	11.4 ± 10.8 (7)	14.0 ± 6.6 (3)
> 50	10.0 (1)	10.0 (1)	—

Within-stratum p-values for NT1 vs NT2 were all > 0.20.

**Table 5b:** Multivariable linear regression for log-transformed diagnostic delay.

Predictor	β (log scale)	95 % CI	p
Age (per year)	+0.020	+0.002 to +0.038	0.032
NT1 vs NT2	−0.059	−0.395 to +0.278	0.735
Female vs Male	−0.241	−0.593 to +0.111	0.184
BMI (per kg/m^2^)	−0.001	−0.025 to +0.022	0.916
ESS (per point)	+0.031	−0.003 to +0.065	0.080
MDD	+0.206	−0.224 to +0.636	0.351
Insomnia	+0.280	−0.254 to +0.815	0.308
Any SDB	+0.180	−0.163 to +0.522	0.308

Dependent variable: log(diagnostic delay + 1). Complete cases n = 85; adjusted R^2^ = 0.119; model F p = 0.022. Log-scale coefficients can be back-transformed as (eβ − 1) × 100 % to express percent change in delay per unit predictor.

### 3.5 Psychiatric comorbidities

Major depressive disorder was documented in 19 patients (16.8 %; 15.4 % in NT1 vs 18.8 % in NT2, p = 0.636); generalized anxiety disorder in 11 (9.7 %; 7.7 % vs 12.5 %, p = 0.524); insomnia disorder in 11 (9.7 %; 7.7 % vs 12.5 %, p = 0.524); and restless legs syndrome in 1 (0.9 %). None of these conditions differed significantly between subtypes. Patients with documented MDD had a non-significantly longer diagnostic delay (median 7.0 vs 4.0 years, Mann–Whitney p = 0.128).

### 3.6 Pharmacological management

Wake-promoting therapy was uniformly high in both subtypes (modafinil: 92.3 % NT1 vs 83.3 % NT2, p = 0.139; methylphenidate: 12.3 % vs 20.8 %, p = 0.221). Anticataplectic and antidepressant prescribing was markedly concentrated in NT1: any antidepressant 73.8 % vs 22.9 % (p < 0.001), venlafaxine 40.0 % vs 6.2 % (p < 0.001), fluoxetine 18.5 % vs 4.2 % (p = 0.023) and clomipramine 10.8 % vs 0 % (p = 0.020). Escitalopram (10.8 % vs 6.2 %, p = 0.513) and imipramine (4.6 % vs 8.3 %, p = 0.456) did not differ significantly (Table 6).

**Table 6:** Pharmacological management by narcolepsy subtype.

Medication	NT1 (n = 65)	NT2 (n = 48)	p
Modafinil, n (%)	60 (92.3)	40 (83.3)	0.139
Methylphenidate, n (%)	8 (12.3)	10 (20.8)	0.221
Venlafaxine, n (%)	26 (40.0)	3 (6.2)	< 0.001
Fluoxetine, n (%)	12 (18.5)	2 (4.2)	0.023
Escitalopram, n (%)	7 (10.8)	3 (6.2)	0.513
Imipramine, n (%)	3 (4.6)	4 (8.3)	0.456
Clomipramine, n (%)	7 (10.8)	0 (0.0)	0.020
Any antidepressant, n (%)	48 (73.8)	11 (22.9)	< 0.001

A subgroup analysis examined whether fluoxetine prescribing in narcolepsy patients reflected mood-disorder treatment rather than narcolepsy-targeted therapy. Among patients receiving fluoxetine (n = 14), only 3 (21.4 %) had documented MDD, and 2 (14.3 %) had GAD; in the NT1-only subgroup (n = 12 on fluoxetine), 8 had neither MDD nor GAD recorded, consistent with fluoxetine being used primarily for cataplexy and REM-intrusion symptoms in this cohort rather than for psychiatric comorbidity. Sodium oxybate, pitolisant, and solriamfetol, recommended in international NT1 guidelines [11,12], were not prescribed because they are not currently on the institutional and Saudi Arabia formulary.

## 4 Discussion

In this 10-year retrospective cohort from a Saudi tertiary sleep center, narcolepsy was characterized by a substantial diagnostic delay that increased with age, a high prevalence of comorbid sleep-disordered breathing in which female sex and younger age, but not BMI, were independent less risk factors, and a pharmacological treatment landscape dominated by modafinil and SNRI/SSRI antidepressants in the absence of newer narcolepsy-specific agents. Three principal themes warrant emphasis.

First, the median diagnostic delay of 5 years (mean 6.6 ± 5.7 years) in our cohort is shorter than the 10.5 years reported in a U.S. cohort [13], 9.7 years in a European multicentre study [14], 8.2 years in an international survey [15], and the > 8 years previously described in Saudi Arabia [4]. Several factors plausibly contribute to this comparatively shorter delay: (i) KFMC is a centralised tertiary sleep-medicine centre with dedicated narcolepsy expertise; (ii) increased awareness of narcolepsy among Saudi primary-care physicians and the public has been promoted in recent years through professional society activity, Ministry of health and Saudi Society of Sleep Medicine; (iii) the cohort skews young (median age 27 years) and a greater proportion of younger patients tends to result in shorter delays in retrospective designs; and (iv) the national tertiary referral pathway effect concentrates higher-suspicion patients as KFMC consider national and biggest referral sleeep center for all MOH hospitlas. Notwithstanding these explanations, a 5-year delay remains substantial and is incompatible with optimal care: it is associated with cumulative academic, occupational, and psychosocial morbidity that is largely preventable.

Second, age was the single independent predictor of diagnostic delay in our cohort (β = +0.020 per year on the log scale, equivalent to a 2.0 % increase in delay per year of age, p = 0.032). Pediatric and adolescent patients (≤20 years) had a median delay of less than 4 years, whereas patients diagnosed in the fifth decade had a mean delay of more than 12 years. This pattern has been reported in other cohorts [14,16] and likely reflects a combination of greater clinical suspicion for narcolepsy in young patients presenting with EDS, attribution of adult-onset sleepiness to lifestyle or psychiatric causes, and recall bias for distant symptom-onset. Importantly, the cardinal symptoms of NT1: cataplexy, sleep paralysis, and hallucinations did not shorten diagnostic delay, suggesting that the recognition pathway for narcolepsy fails not only when symptoms are subtle (NT2) but also when they are classical (NT1). The Epworth Sleepiness Scale was an independent predictor of being delayed diagnostically (≥5 years; aOR 1.15 per point, p = 0.013), indicating that the sleepiest patients accumulate the longest delays, a counter-intuitive finding that probably reflects misattribution of high ESS to OSA, depression, or lifestyle factors before MSLT is performed.

Third, sleep-disordered breathing was present in 46.0 % of the cohort (any-SDB), with REM-OSA in 40.7 %. These figures are slightly lower than those reported by Dhafer and colleagues in another Saudi narcolepsy cohort in OSA general but higher in REM-OSA (OSA 55.8 %; REM-OSA 26.4 %) [9] but substantially exceed both general population estimates from Saudi Arabia (overall OSA prevalence ∼ 8.8 % [7]) and international estimates of OSA in narcolepsy in earlier series (25–33 % [17,18]). Hoshino et al. found OSA in 39.7 % of adult narcoleptic patients, with REM-OSA in 30–43 % of those with OSA [8], values that bracket our findings. Importantly, our multivariable analysis identified female sex as independently protective (aOR 0.34) and increasing age as an independent risk factor (aOR 1.05 per year). BMI did not retain independent significance, likely because the Saudi background prevalence of overweight/obesity is high and confounds the OSA–BMI relationship at the cohort level. The sex effect is consistent with both general OSA epidemiology [7] and prior narcolepsy literature [19], and supports a low threshold for PSG in adult male narcolepsy patients.

### 4.1 Psychiatric comorbidity and the diagnostic pathway

Psychiatric comorbidity was prevalent: MDD in 16.8 % and GAD in 9.7 % of patients, with no statistically significant differences between NT1 and NT2. These rates are broadly consistent with population-based samples of patients with narcolepsy reporting MDD in 17–19 % [15,20], slightly below international averages. Although univariate analysis suggested that MDD was associated with a non-significant trend toward longer delay (median 7 vs 4 years, p = 0.128), this did not remain significant after multivariable adjustment for age. The clinically important implication is that overlap between psychiatric symptoms and narcolepsy-related sleepiness may divert evaluation toward mood explanations, and systematic screening for narcolepsy in patients with refractory or atypical EDS attributed to depression is warranted.

### 4.2 Pharmacological management

Modafinil dominated wake-promoting therapy across both subtypes (88.5 % overall), consistent with international guidelines [11] and reflecting both the local formulary and the agent's favorable risk–benefit profile. Anticataplectic and SSRI/SNRI prescribing were strongly concentrated in NT1, with venlafaxine the most commonly used SNRI (40 % of NT1), paralleling the cataplexy burden of NT1 and the established evidence base for serotonin-norepinephrine reuptake inhibitors [2,11]. Fluoxetine was prescribed at a higher rate in NT1 (18.5 %) than in NT2 (4.2 %; p = 0.023). Subgroup analysis showed that fluoxetine use was largely independent of documented MDD or GAD in NT1 patients, consistent with use for cataplexy or hallucinations rather than for psychiatric comorbidity per se.

Sodium oxybate, pitolisant, and solriamfetol, which are strongly recommended for NT1 management by the American Academy of Sleep Medicine [11] and the European Academy of Neurology/European Sleep Research Society [12], were not used in this cohort because none is currently on the KFMC and MOH formulary. As regulatory approval and access expand in the Gulf region, prospective data on the efficacy and safety of these agents in Saudi narcoleptic patients will be essential.

### 4.3 Strengths and limitations

Strengths of this study include the largest single-center narcolepsy cohort reported from Saudi Arabia to date, strict ICSD-3 ascertainment, in-laboratory PSG with MSLT for all participants, and a 10-year window that captures changes in clinical practice. Several limitations should be acknowledged. First, the retrospective design is subject to information bias, particularly in patient-recalled symptom onset, which contributes to the 20 % missingness in diagnostic delay. Second, CSF hypocretin-1 measurement and HLA-DQB1*06:02 genotyping were not performed; therefore, NT1/NT2 classification depended on clinical cataplexy and MSLT rather than biomarker confirmation. Third, sleep architecture data beyond AHI, REM-AHI, and sleep efficiency (stage percentages, REM latency, MSLT mean sleep latency, SOREMP counts) were not consistently captured in the structured EMR fields used for this analysis; these variables are a priority for prospective re-abstraction. Fourth, the single-center design limits generalisability to other regions of Saudi Arabia. Fifth, sodium oxybate, pitolisant, and solriamfetol were not used due to formulary constraints; therefore, this cohort cannot inform the use of these newer agents in the Saudi context. Finally, the modest sample size limits the statistical power of sub-stratified models, particularly in the > 50-year age stratum (n = 1).

## 5 Conclusion

In this Saudi tertiary-center narcolepsy cohort, age at presentation was the dominant determinant of diagnostic delay, sleep-disordered breathing was highly prevalent and was independently associated with male sex and older age, and the pharmacological treatment landscape was dominated by modafinil and SNRI/SSRI antidepressants in the absence of newer narcolepsy-specific agents. Recognition pathways must be strengthened, particularly for adult and middle-aged patients with chronic EDS attributed to OSA, depression, or lifestyle, and access to oxybate, pitolisant, and solriamfetol should be expanded.

## CRediT authorship contribution statement

**Mana Alshahrani:** Writing – review & editing, Writing – original draft, Visualization, Validation, Supervision, Software, Resources, Project administration, Methodology, Investigation, Funding acquisition, Formal analysis, Data curation, Conceptualization. **Nora Bedaiwi:** Writing – review & editing, Writing – original draft, Visualization, Validation, Data curation. **Suhaila Aljawder:** Writing – review & editing, Writing – original draft, Visualization, Validation, Data curation. **Riyad Allehebi:** Writing – review & editing, Writing – original draft, Visualization, Validation, Methodology, Data curation. **Ahmed Abdelbasit:** Writing – review & editing, Writing – original draft, Visualization, Validation, Data curation. **Alyah A. Alshehri:** Writing – review & editing, Writing – original draft, Visualization, Software, Resources, Data curation. **Sumayyah I. Fallatah:** Writing – review & editing, Writing – original draft, Visualization, Validation, Investigation, Formal analysis, Data curation. **Omaima T. Alomani:** Validation, Resources, Investigation, Data curation. **Lama A. Alhomayin:** Writing – original draft, Visualization, Validation, Data curation. **Talal N. AlHoshan:** Writing – original draft, Investigation, Data curation.

## Data availability

The de-identified dataset is available from the corresponding author upon reasonable request and with institutional approval.

## Ethics

The protocol was approved by the KFMC Institutional Review Board (approval no. 26-131). Informed consent was waived because of the retrospective design and the use of de-identified data.

## Funding

This research received no specific grant from any funding agency in the public, commercial, or non-profit sectors.

## Declaration of competing interest

The authors declare that they have no known competing financial interests or personal relationships that could have appeared to influence the work reported in this paper.
