# Supplementary material for: Sleep-disordered breathing and psychiatric comorbidities in narcolepsy: A 10-year retrospective study from Saudi Arabia
Source: Sleep Med X. 2026 Sep 6;12:100199. doi: 10.1016/j.sleepx.2026.100199 (PMC13583604; doi:10.1016/j.sleepx.2026.100199)
Supplement: Supplementary file 1 [file mmc1.docx]

**Supplementary Material**

*Manuscript SLEEPX-D-26-00151 Diagnostic Delay, Sleep-Disordered Breathing, and Psychiatric Comorbidities in Narcolepsy: A 10-Year Retrospective Study from Saudi Arabia*

Corresponding author: Dr Mana Alshahrani · dr.mana1986@hotmail.com

# Supplementary Figure S1 · Forest plot : Multivariable predictors of any sleep-disordered breathing

*Supplementary Figure S1. Adjusted odds ratios (95 % confidence interval) from the multivariable logistic regression model of any sleep-disordered breathing (SDB) presented in Table 4 of the main text. Reference categories: NT2 for narcolepsy subtype, male for sex. Complete-case analysis n = 99. Female sex was independently protective (adjusted OR 0.34, 95 % CI 0.13–0.86, p = 0.023) and older age increased the odds of any SDB (adjusted OR 1.05 per year, 95 % CI 1.00–1.11, p = 0.037). Narcolepsy type, body mass index and Epworth Sleepiness Scale did not show independent associations.*

# Supplementary Figure S2 · Sex differences in sleep-disordered breathing prevalence

*Supplementary Figure S2. Prevalence of obstructive sleep apnea (OSA, AHI ≥ 5/h), REM-related OSA, and any sleep-disordered breathing (any-SDB) among male and female patients in the cohort. Men had a markedly higher prevalence across all three definitions (OSA 50.0 % vs 17.9 %, p = 0.001; REM-OSA 50.0 % vs 23.1 %, p = 0.006; any-SDB 55.4 % vs 28.2 %, p = 0.006).*

# Supplementary Table S1 · Age-stratified sleep-disordered breathing and psychiatric comorbidities

This table is identical to Table 3 of the main manuscript and is duplicated in the Supplementary Material for readers who wish to access the age-stratified data separately.

| **Age group** | **n** | **Any SDB** | **OSA (AHI ≥ 5/h)** | **REM-OSA** | **AHI, median (IQR)** | **MDD** | **GAD** |
| --- | --- | --- | --- | --- | --- | --- | --- |
| ≤ 20 | 29 | 11/29 (37.9%) | 8/28 (28.6%) | 11/28 (39.3%) | 2.5 (1.0–5.5) | 4/29 (13.8%) | 1/29 (3.4%) |
| 21–30 | 39 | 13/39 (33.3%) | 12/38 (31.6%) | 11/38 (28.9%) | 3.6 (1.7–6.6) | 5/39 (12.8%) | 3/39 (7.7%) |
| 31–40 | 29 | 17/29 (58.6%) | 14/28 (50.0%) | 14/28 (50.0%) | 5.0 (2.1–13.0) | 7/29 (24.1%) | 5/29 (17.2%) |
| 41–50 | 15 | 10/15 (66.7%) | 9/15 (60.0%) | 9/15 (60.0%) | 6.9 (2.4–8.9) | 3/15 (20.0%) | 1/15 (6.7%) |
| > 50 | 1 | 1/1 (100%) | 1/1 (100%) | 1/1 (100%) | — | 0/1 (0%) | 1/1 (100%) |

*Age-related trend for any-SDB: logistic-regression odds ratio 1.06 per year of age (95 % CI 1.02–1.11, p = 0.007). AHI = apnea–hypopnea index; MDD = major depressive disorder; GAD = generalised anxiety disorder; SDB = sleep-disordered breathing.*
